# Comorbidities in Spondyloarthritis

**DOI:** 10.3389/fmed.2018.00062

**Published:** 2018-03-12

**Authors:** Anna Moltó, Elena Nikiphorou

**Affiliations:** ^1^Rheumatology B Department, Hôpital Cochin, Assistance Publique-Hôpitaux de Paris, Paris, France; ^2^INSERM (U1153) PRES Sorbonne Paris-Cité, Paris, France; ^3^Academic Rheumatology Department, King’s College London, London, United Kingdom; ^4^Department of Rheumatology, Whittington Hospital, London, United Kingdom

**Keywords:** spondyloarthritis, comorbidity, cardiovascular, osteoporosis, infection, cancer

## Abstract

Comorbidities in spondyloarthritis (SpA) add to the burden of disease by contributing to disease activity, functional and work disability, and mortality. Thus, awareness of comorbidities in SpA is crucial to improve their screening and management and to ultimately improve outcomes in those affected. Osteoporosis has been reported to be the most prevalent comorbidity in SpA, and its risk is increased in these patients, compared with the general population; the risk of vertebral fractures requires further evaluation. Cardiovascular risk is also increased in this population, both due to an increase of the traditional cardiovascular risk factors in these patients, but also due to the presence of inflammation. The role of non-steroidal anti-inflammatory drugs in this increased risk needs further elucidation, but there is consensus on the need to encourage smoking cessation and to perform periodic evaluation of cardiovascular risk in these patients, particularly in the case of change in treatment course. Concerning the risk of cancer, no increased risk inherent to SpA seems to exist. However, an increased neoplastic risk can occur due to SpA treatments, e.g., P-UVA. Data are sparse on the risk of infections compared with rheumatoid arthritis, but there appears to be no risk in the absence of TNF-inhibitor exposure. Regardless of which comorbidity, a gap exists between recommendations for their management and actual implementation in clinical practice, suggesting that there is still a need for improvement in this area. Systematic screening for these comorbidities should improve both short- and long-term outcomes in SpA patients.

## Introduction

The term “spondyloarthritis” (SpA) encompasses inflammatory rheumatic diseases affecting mainly the axial skeleton. In SpA, extra-axial manifestations can be seen, namely, enthesitic and peripheral articular manifestations, but also extra-articular manifestations, such as skin, eye, or gut involvement [psoriasis, uveitis, and inflammatory bowel disease (IBD), respectively] (1). Apart from these manifestations, which are directly related to SpA patients may also suffer from other distinct additional entities, classically referred to as “comorbidities”; the latter may have existed or may occur during the clinical course of a patient who has the index disease under study (2, 3). This is unlike extra-articular manifestations, which may occur during the course of the disease and belong to the spectrum of SpA but do not by definition fulfill the criteria for comorbidity.

Most of the evidence on the impact of comorbidities in rheumatic diseases comes from rheumatoid arthritis (RA), where the presence of comorbidity has been found to be a more significant predictor of premature death than shared epitope, rheumatoid factor, or erosions (4). Although less-well studied in SpA, evidence is increasing on the prevalence of several comorbid conditions (5) and their added burden on excess disease activity, functional disability, poor work-related outcomes (6), and mortality (7). Thus, it is crucial to increase awareness on comorbidities, especially those more frequently associated with the disease and/or its treatment. This way, their screening and management can be improved, ultimately resulting in better outcomes for these patients.

The focus of this review will be on four key disease areas that have been observed in SpA patients, focusing mainly in the axial forms of the disease, i.e., axial spondyloarthritis (axSpA), but also, for some of the comorbidities we will also present data from the peripheral forms of SpA [e.g., peripheral SpA or psoriatic arthritis (PsA)]: osteoporosis, cardiovascular disease (CVD), cancer, and infections (5). Although some of these conditions, especially the former two listed above, have been referred to in literature as complications of the underlying index disease, here we refer to them as comorbidities. In this review, we will also make the distinction between these conditions or comorbidities and related risk factors for these (i.e., hypertension, dyslipidemia, diabetes mellitus, or smoking for CVD).

## Osteoporosis

### Osteoporosis in SpA

Bone formation is the cornerstone lesion in axSpA, which leads to ankylosis and permanent disability of patients; paradoxically, osteoporosis or low bone density has been found to be the most prevalent comorbidity in these patients (5). Osteoporosis in RA has been largely documented, both related to the phenotype of RA patients (postmenopausal women), to glucocorticoid treatment (very frequently used in RA and well-known osteoporosis inducer), but also related to inflammation (8, 9). Indeed, several studies have highlighted that a better control of disease activity (i.e., inflammation) leads to lower rates of bone loss in RA patients (10, 11). Osteoporosis in SpA patients can be hardly explained by the phenotypic characteristics such as age and gender, or systemic treatments, since usually the disease occurs in young males (1) and glucocorticoids are barely used, particularly in axial forms, unless they present with concomitant IBD (12).

Osteoporosis in axSpA, particularly in long-standing forms, can be related to ankylosis and immobilization: indeed, prevalence of osteoporosis in radiographic axSpA (r-axSpA) patients has been reported to range between 19 and 50% (13, 14) and disease duration and ankylosis of the spine [e.g., measured by the modified Stokes Ankylosing Spondylitis Spine Score (mSASSS)] have been classically found to be predisposing factors: in a study including 80 patients with r-axSpA with a mean disease duration of 10.8 years, disease duration was significantly and independently associated with osteoporosis [odds ratio (OR) = 1.13, 95% confidence interval (CI): 1.03–1.25], while body mass index (BMI) was found to be inversely associated (OR = 0.82, 95% CI = 0.69–0.93) (14). Another study including 204 r-axSpA patients and a mean disease duration of 15 ± 11 years reported that low BMD was associated with older age, disease duration, mSASSS, and Bath Ankylosing Spondylitis Metrology Index; furthermore, mSASSS significantly inversely correlated with lumbar bone mass density (BMD) (*r* = −0.389, *P* < 0.001) (15).

However, osteoporosis has also been reported in early forms of the disease and thus cannot be related only to spine ankylosis and immobilization (16). Briot et al. reported a prevalence of osteoporosis of 13.0% in a sample of 332 patients with early SpA (less than 3 years duration) from the DESIR cohort. Interestingly, the factors associated with osteoporosis were inflammation, systemic (increased ESR or CRP, OR = 2.60, 95% CI = 1.06–6.35) or local, defined by bone marrow edema (inflammatory lesions) on MRI (OR = 4.63, 95% CI = 1.90–11.31) and male gender (OR = 9.60, 95% CI = 2.73–33.78) (17).

Inflammation has been linked to increased bone resorption and impaired bone formation, by inflammatory mediators’ action on osteoclast activity. Thus, there is a rationale to suggest that inflammation has an unfavorable effect on bone remodeling, and is the basis of the use of potent anti-inflammatory drugs to protect bone.

### Fractures in SpA

#### Vertebral Fractures (VFs)

The prevalence of VFs in SpA patients is controversial: it has been classically reported that 30–40% patients with SpA present with VFs (18) and have greater risk of VFs (ranging from threefold to sevenfold) when compared with the general population (19, 20). Actually, there is concern regarding the definition of VFs across studies, since patients with SpA can present with vertebral deformities, particularly at the dorsal spine (e.g., due to lesions at anterior corners, wedging secondary to inflammatory lesions and hyperkyphosis). Semiautomated methods of morphometry are often used to assess VFs in large studies, and these methods might also capture these vertebral deformities, overestimating the prevalence of osteoporotic VFs in this population, since not all vertebral deformities are VFs (21, 22).

Incidence of VFs in a 4-year prospective study including in 298 r-axSpA patients 13.6% at 4 years, and risk factors included the presence of VFs at baseline and the presence of elevated CRP (23). Conversely, a very recent analysis of the incidence of VFs in an early axSpA population (the DESIR cohort) of 433 patients prospectively followed for 5 years and with X-rays available both at baseline and at 5 years revealed only seven incident VFs over the 5 years of follow-up, i.e., a 5-year incidence of VFs of 1.6%. In this recent study, assessment of VFs was not performed by any semiautomated method, but by an expert central reader (24).

Several factors have been associated with VFs in SpA; for example, mSASSS has been classically associated with VFs in SpA, probably related to lower BMD in patients with ankylosing disease (and therefore more disabled and less active) (14) but also due to the potential difficulties with peripheral vision secondary to limited range of spinal mobility and consequently higher risk of falls. Disease duration and hyperkyphosis have been reported as risk factors for VFs (8). SpA patients with VFs have lower BMD than patients without, and femoral neck is the best discriminant site, but low BMD does not seem sufficient for to prediction of fracture in this population (25).

#### Spinal Fractures

Spinal fractures should be distinguished from VFs, since they are not related to osteoporosis or low BMD and are the consequence of trauma (frequently minor traumatisms) in patients with an ossified spine. This event is a major complication in SpA, but is not strictly considered comorbidity, and thus its prevalence and mechanisms will not be detailed in this review.

### Osteoporosis and SpA Treatments

#### Non-Steroidal Anti-inflammatory Drugs

Since in SpA inflammation leads to stiffness and loss of mobility, anti-inflammatory drugs are expected, through both the increased mobility related to pain relief and the increased activity, to have an effect on BMD. In a prospective analysis of the early SpA DESIR cohort of 265 patients (54% male, mean age 34.4 years) who had BMD measurements at baseline and at 2 years, use of non-steroidal anti-inflammatory drugs (NSAIDs) had protective effects on hip bone loss in patients (OR = 0.09, 95% CI = 0.02–0.50) (26). This effect has also been observed for VFs: in a primary care-based nested case–control study, including patients with SpA, the risk of any clinical fracture was decreased in patients taking NSAIDs (OR = 0.65, 95% CI = 0.50–0.84) (25). However, conflicting results have been also reported, with an excess risk of any clinical fracture in patients with SpA, and even a higher risk in patients using NSAIDs, probably because these patients had a more severe disease and thus, a higher utilization of NSAIDs, since these latter data are issued from claim databases (27). Thus, the effect of NSAIDs on VF prevention needs to be further explored.

#### Biologic DMARDs

The positive effect of TNF inhibitors (TNFi) on BMD in SpA patients has been reported in several prospective studies in patients: a significant increase in BMD 2.3 and 11.8% was reported in a follow-up study of 106 SpA patients at 2 and 6 years, respectively (28, 29). This effect has also been confirmed in early forms of the disease (DESIR cohort) where the analysis of the 265 patients with BMD available over the 2-year follow-up, TNFi use was significantly and independently protective for bone loss (OR = 0.43, 95% CI = 0.20–0.93) (26). Furthermore the beneficial effect of TNFi has been confirmed by a recent meta-analysis of longitudinal trials and one RCT, with a total of 568 r-axSpA patients (mean disease duration of years): lumbar spine BMD increased by 8.6% (95% CI = 6.8–10.3%, *P* < 0.00001) after 2 years (30).

No clinical data are available yet regarding the potential positive effects on bone of IL-17 blockade with IL-17 inhibitors used in SpA treatment, but animal models seem to confirm this hypothesis (31).

Although no specific guidelines for the management of osteoporosis in SpA exist, some national scientific societies have proposed to perform a BMD evaluation at least once in the course of the disease in patients with SpA (5). However, in patients with severe osteoporosis, prevalent fractures or several risk factors, available guidelines for osteoporosis management (e.g., male osteoporosis) should be used (8).

In summary osteoporosis is a highly prevalent comorbidity in SpA, higher than the general population; however, risk of VF requires further evaluation, as current prevalence and incidence reported in the literature might have been over-estimated by the automate and semiautomate methods of evaluation of such VFs. Nevertheless, these findings support that measurement of BMD should be performed at least once during the disease course.

## Cardiovascular Disease

An increase in mortality has been reported in patients with axSpA (7, 32, 33). Indeed, a recent study reported an age-adjusted and sex-adjusted mortality hazard ratio (HR) of 1.60 (95% CI = 1.44–1.77), with increased mortality for men [age-adjusted HR = 1.53 (1.36–1.72)] and women [age-adjusted HR = 1.83 (1.50–2.22)] in patients with axSpA and CVD accounted for 34.7% of all deaths (33). Indeed, CVD is consistently found as the leading cause of mortality in patients with axSpA (7, 33–37), with percentages ranging from 30 to 50% of all-cause deaths in this population (7). This increased mortality can be explained both by an increase in the prevalence of CVD in axSpA but also by an increase in cardiovascular risk factors (CVRFs) compared with the general population. This increase in cardiovascular mortality was the rationale to include SpA patients in the scope of the 2015/2016 EULAR recommendations for cardiovascular risk management, as well as in other recommendations (38, 39).

### Ischemic Heart Disease (IHD) and Stroke

A recent cross-sectional study including 3,984 patients with SpA [mean age 44 (13)] found a prevalence of IHD and stroke of 2.7% (95% CI = 2.2–3.2) and 1.3% (95% CI = 0.9–1.7), respectively (5). A meta-analysis (36) and some more recent prospective studies (40, 41) have consistently reported increased risk of CVD in patients with axSpA: the 2011 meta-analysis (36) reported an increase risk of IHD (OR = 1.60, 95% CI = 1.32–1.93) and stroke (OR = 1.50, 95% CI = 1.39–1.62) in patients with r-axSpA, compared with controls (36). A more recent prospective study including patients with axSpA found an increased risk of IHD in patients with r-axSpA and not radiographic axSpA (nr-axSpA) [age- and sex-adjusted HR = 1.54 (95% CI = 1.31–1.82) and HR = 1.36 (95% CI = 1.05–1.76), respectively] and also for stroke but only for r-axSpA [age- and sex-adjusted HR = 1.25 (95% CI = 1.06–1.48) and HR = 1.16 (95% CI = 0.91–1.47), for r-axSpA and nr-axSpA, respectively], compared with the general population (41).

### Cardiovascular Risk Factors

#### Classic CVRFs

There are five classic modifiable CVRFs (hypertension, smoking, dyslipidemia, diabetes, and obesity), and they are estimated to account for more than 50% of all CV deaths (42). In this review, we will not discuss the non-modifiable CVRF (e.g., age, gender, or family history of CVD).

##### Hypertension

Hypertension is one of the most prevalent CVRF, estimated to account for 50% of all strokes and IHD events in the general population (43). Several studies have reported an increased prevalence of hypertension in SpA patients, compared with controls both in axial and peripheral forms of SpA (including PsA) (5, 37, 44–46): a recent cross-sectional international study including 4,000 SpA patients worldwide (5) reported hypertension to be the most frequent CVFR in this population, with a prevalence to 33.5% (95% CI = 32.0–35.0%) patients; another study (45) reported a substantially higher prevalence of hypertension in patients with r-axSpA compared with the general population in the Netherlands (41 vs. 31%, respectively).

The mechanisms underlying this increased prevalence of hypertension in SpA are likely to be multiple: first, hypertension in patients with SpA has been found to be associated with disease activity (47), which might be due to increased inflammatory pathways (48) but also potentially to decreased mobility due to stiffness leading to increased sedentarism in patients with greater disease activity. Furthermore hypertension seems to be underdiagnosed in patients with SpA: the COMOSPA study reported that systematic screening for hypertension revealed an increased systolic blood pressure in 14.7% of the SpA patients without any known history of hypertension (5).

##### Cigarette Smoking

Prevalence of cigarette smoking in the general population has been recently reported to be around 15% (data from the US) (49); this prevalence appears to be increased in patients with axSpA, with reported numbers up to 30 and 40% (5, 50). Furthermore, smoking has been associated with increased acute phase reactants [high sensitivity CRP (51)], but also, in the specific case of axSpA, with increased disease activity and structural progression (52–54); therefore, smoking in axSpA patients would have a double role, as traditional CVRF but also increasing disability and ankylosis, which might potentially lead to a more sedentary lifestyle, another very well-known traditional CVRF.

##### Dyslipidemia

Prevalence of dyslipidemia in the general population is estimated to be 12% in adults above 20 years and is significantly increased in patients with premature IHD, i.e., up to 80% in those patients (55). A paradoxical decrease in lipids (e.g., total cholesterol, LDL cholesterol, and HDL cholesterol) has been reported in patients with other rheumatic inflammatory diseases, in particular RA (56). However, while most of the studies have also reported a decrease in HDL cholesterol, a well-known protective factor for CVD (57), in patients with r-axSpA compared with controls, in particular in the presence of active inflammatory disease (37, 46, 58–60), other studies have reported increased total and LDL cholesterol in patients with r-axSpA and PsA (61, 62). Furthermore, some recent studies have reported an impaired endothelial function of HDL in patients with r-axSpA, leading to lower antiatherogenic properties (63). Thus, the role of dyslipidemia in the CV risk in SpA needs further investigation.

##### Diabetes Mellitus

The overall prevalence of diabetes in adults has increased from 4.7% in 1980 to 8.5% in 2014 (64). Diabetes is associated with CVD, and some studies have reported diabetes to be accountable for 10% of the population-attributable risk of a first myocardial infarction (65).

Several studies have suggested an association between diabetes and peripheral forms of SpA (e.g., PsA), with an increased risk of 1.4 (95% CI = 1.3–1.5) for diabetes among patients with PsA compared with patients without rheumatic diseases (66, 67). These differences might be due to the different treatments used in peripheral vs. axial forms, since glucocorticoids, a know risk factor for diabetes, are more often used in peripheral forms and are not recommended in axial forms (12).

##### Obesity

Prevalence of obesity (defined as a BMI above 30 kg/cm^2^) in the general population has been estimated to range between 13% [Europe (68)] and 37% [US (69)]. Obesity is associated with a number of risk factors for CVD, including hypertension, diabetes, and dyslipidemia (70), but other studies have reported BMI to independently predict the occurrence of IHD and stroke after adjusting for traditional risk factors, suggesting a continuous linear relationship between higher BMI and greater risk of CVD (71, 72).

Obesity is not that frequent in pure axial forms of the disease (73). However, prevalence of obesity is increased in patients with peripheral forms of SpA (e.g., PsA) and has been reported to involve to 30% of patients with PsA. In contrast to what has been reported in RA, i.e., RA patients with low BMI (<20 kg/cm^2^) might have greater CV risk due to differences in body composition (56), BMI-CV risk relationship in patients with PsA seem to be similar to the one observed in the general population.

#### New CVRFs

It has been established that inflammation is associated with an increased risk of atheroma development (56), and while systemic inflammation is less often present in axSpA, several studies have reported increased inflammatory markers such as ultrasensitive CRP, IL-6, and homocystein (58, 74). Furthermore, several studies have observed an increased Carotid intima-media thickness, arterial stiffness and endothelial dysfunction (known CVRFs) in patients with axSpA, compared with controls (34, 35, 75, 76).

### CVD and Treatments in SpA

#### Non-Steroidal Anti-inflammatory Drugs

Non-steroidal anti-inflammatory drugs remain the cornerstone treatment in axSpA (12), and these drugs have been reported to increase CVD risk in the general population (77). However, this effect is not apparently found in patients with SpA, and several studies have reported a lack of increase (78) or even a reduction of CVD rates in SpA patients treated with NSAIDs (37, 79). The hypothesis for these results is that inflammation would greatly contribute to the CV burden, and NSAIDs would have a beneficial effect by controlling such inflammation. However, it cannot be completely excluded that these results do not simply highlight a different NSAID prescription rate in patients with previously known CVRFs (prescription bias) although some studies did apply statistical methods to overcome this bias (e.g., propensity score adjustment).

#### Glucocorticoids

Glucocorticoid use is associated with increased CV risk, particularly of IHD (80). Indeed, doses ≥7.5 mg/day of prednisone in several population-based studies have been reported to increase the CV risk (including CV mortality), with a risk ratio ranging from 2.6 to 7.4 (80, 81). However, data support that below this threshold, the risk for CV events seems low, and glucocorticoids are very effective at treating inflammation, which relates to cardiovascular risk (82).

#### Conventional Synthetic DMARDs

Sulfasalazine is the recommended DMARD to be used in peripheral forms of Ref. (12), and there are only few reports suggesting an effect on reducing CV risk (83). This effect might be explained by the inhibitory action of sulfasalazine and its metabolites of arachidonic acid-induced platelet aggregation, which has been reported to be comparable to that achieved by aspirin, a well-known cardioprotective agent (84). Nevertheless in clinical practice, methotrexate if often used for the treatment of peripheral SpA, and greater evidence supports the cardioprotective role of methotrexate in RA (85, 86).

#### Biologic DMARDs

TNF inhibitors are the most widely used biologics in SpA. TNFi have been proven to reduce inflammation, which has been associated with increased CV risk, and several studies have reported to reduce subclinical atherosclerosis in SpA patients treated with TNFi (86–89). Also, TNFi have been reported to increase weight and abdominal fat mass (90), total cholesterol, HDL cholesterol, and LDL cholesterol in patients with axSpA (91), although these changes probably reflect a normalization in these parameters secondary to inflammation control.

IL-17 inhibitors have not been reported to be associated with CV risk, but studies are sparse (92, 93). More conflicting data have been published regarding IL12/23 inhibition, since significantly more major CV events were reported in the active-treatment arms of phase-3 trials in patients with psoriasis (94), while prospective long-term observational data have not found an increased CV risk (95, 96).

In summary, both CV mortality and morbidity seem to be increased in SpA, both related to an increased prevalence of most of the classic CVRFs, but also linked to inflammation. This forms a strong rationale for the systematic evaluation of CV risk in all SpA patients, at least every 5 years or more frequently in the case of a change in the treatment course, as recommended by EULAR and other societies (38, 39).

## Cancer

### Cancer and SpA

While an increased risk of malignancy (i.e., lymphoma) has been reported in patients with RA (97), no increased risk of cancer has been reported in patients with SpA (98, 99). Several registers have consistently reported reassuring data: Swedish registers reported a standardized incidence ratio (SIR) of malignancy of 1.05 (95% CI = 0.94–1.17), for the 1965–1995 period in patients with axial SpA (79); a more recent collaborative analysis of two Scandinavian registers confirmed this findings for the 2001–2011 period, with an RR for malignancy of 1.1 (1.0–1.2) in SpA patients compared with the general population, and very similar results for the r-axSpA and PsA forms [RR = 1.1 (95% CI = 1.0–1.3) and RR = 1.0 (0.9–1.1), respectively] (100). A Canadian prospective cohort of PsA also confirmed these findings, reporting a non-significant malignancy SIR of 0.98 (95% CI = 0.77–1.24) (101).

#### Colorectal Cancer (CRC) and SpA

The risk of developing a CRC is increased in patients with IBD, which often coexists with SpA: this risk is estimated to be twofold increased in this population, particularly in males (RR = 1.6, 95% CI = 1.2–2.2 vs. RR = 1.9, 95% CI = 1.5–2.4 for males vs. females, respectively) (102).

However, prospective observational data from SpA registers and cohorts have not reported an increased risk of CRC: indeed, in the Swedish register no increased risk for colon cancer was observed (SIR 0.95, 95% CI = 0.58–1.47), and the risk of rectal cancer was found to be significantly less frequent (SIR = 0.41, 95% CI = 0.15–0.89) (103). This latter finding for rectal cancer has not been confirmed in other registers, but no increased risk has been reported either (101).

Screening recommendations for the most common type of cancer are available for the general population and some are specific depending on the treatment (e.g., dermatology visit in patients receiving TNFi treatment), but their implementation has been reported to be far from optimal: a cross-sectional international study (5) revealed that SpA patients were in agreement with general population recommendations for cancer prevention in 32.7% (CRC) to 44.0% (breast cancer) of patients at risk; and that only 10.7% of patients with TNFi treatment were optimally screened for skin cancer.

### Cancer and SpA Treatments

#### Radiation Therapy and Cancer

Historically, an increase in malignancy risk and mortality was reported in patients with SpA (particularly in axial forms) due to the historic treatment for SpA, which was based on radiotherapy of the spine: an increased mortality risk up to 28% was reported in SpA patients undergoing this treatment, compared with the general population, and a particular threefold increase in mortality due to leukemia in these patients (104). Fortunately, with the arrival of novel and effective therapeutic options, radiotherapy courses have been abandoned for the treatment of SpA.

#### Phototherapy for Skin Psoriasis and Cancer

Oral 8-methoxypsoralen-UV-A (P-UVA) and narrowband UVB are phototherapies used in skin psoriasis. An increased skin cancer risk [squamous cell carcinoma (SCC) mainly among the non-melanoma cancers] has been reported in patients undergoing P-UVA therapy, with a dose-ranging effect: the risk of SCC was significantly higher for patients exposed to >200 P-UVA, compared with low-dose exposed patients (<100 sessions), while results for melanoma were conflicting, with most of the US studies suggesting an increased risk of melanoma in exposed patients, while European studies did not find any association (105).

#### NSAIDs and Cancer

The potential beneficial effect of NSAIDs in recurrent CRC has been reported in systematic reviews: in particular, COX-2 inhibitors (celecoxib and rofecoxib) were reported to be highly effective in reducing the incidence of recurrent colorectal adenomas (106, 107): in a recent meta-analysis, the incidence of recurrent adenomas and advanced adenomas over a 3-year follow-up was significantly reduced [pooled RR = 0.66, 95% CI = 0.59–0.72 for celecoxib and RR = 0.76 (0.69–0.83) for rofecoxib]. However, the increased risk for gastrointestinal events and the relative contra indication in patients with IBBD (the main risk population) represents a crucial drawback to the use of these drugs as prevention therapy in populations at risk. In any case, no data suggest that NSAIDs may increase the risk of CRC.

#### TNFi and Cancer

Most of the data on TNFi and cancer are derived from RA trials and have not reported any increased risk for malignancy (108–110) although some studies present conflicting data, mainly for lymphoma which is inherently increased in RA (111, 112) and skin cancer (113, 114) as already mentioned. Compared with the literature on cancer in RA patients, treated with TNFi, data on cancer risk in SpA patients exposed to TNFi are limited, and most of the available studies only include RCTs (i.e., short follow-up) (115–117); nevertheless, none of them report an increased risk for malignancy in this population.

A recent collaborative analysis of two Scandinavian registers including 8,703 patients with SpA initiating a first TNFi between 2001 and 2011 reported no increased cancer risk in TNFi exposed SpA patients [compared with TNFi-naïve SpA patients, RR = 0.8 (95% CI = 0.7–1.0)]. Similar results were found for r-axSpA and PsA, when analyzed separately [RR = 0.8 (95% CI = 0.6–1.1) and RR = 0.9 (95% CI = 0.7–1.1), for r-axSpA and PsA, respectively] (100). Data on the increased risk of a second neoplasm in patients with history of cancer and treated with TNFi are controversial with some studies suggesting a greater risk in TNFi SpA treated patients compared with the general population (118), while larger studies focusing in RA have not found any significant increased risk (119, 120). Due to the small number of studies evaluating this subgroup of patients with previous history of cancer, these treatments should be used with caution, and always in agreement with the oncological team.

In summary, it does not seem that SpA patients are at greater risk of cancer compared with the general population, except for those exposed to some treatment modalities (e.g., P-UVA therapy); data on TNFi are controversial but no increased risk appears to exist in SpA, in contrast to RA.

## Infection

### Infection and SpA

In contrast to RA, data on infectious risk are poor and derive mainly from randomized controlled trials and are therefore issued from selected populations and with a short-follow-up. A 2008 systematic review and meta-analysis including 14 RCTs reported only two serious infections in 2,202 r-axSpA patients not exposed to immunosuppressive drugs [0.09%, i.e., 0.4 per 100 person years (pyrs)] (121). Conversely, data from observational trials yield slightly higher severe infection rates: a recently published analysis of 440 SpA patients followed for a total of 1,712 patient years (pys) revealed 23 serious infections, i.e., a serious infection rate of 1.3 (95% CI = 0.9–2.0)/100 pys (122). Interestingly, in this study, the use of DMARDs, but not specifically the use of TNFi was associated with infection.

### Infection and TNFi

In most studies, infection rates are higher in the group of patients exposed to TNFi compared with placebo or any other csDMARD: in the 2008 systematic review (121), 14 serious infections were found in the TNFi exposed group (14/996, 0.7% 95% CI = 0.3–1.4%), i.e., 1.9/100 pyrs, but the meta-analysis of the RCTs showed that the increase in serious infections with TNF blockers compared with placebo was not significant: risk difference = 0.4% (−8 to 1.6%). However, another meta-analysis of etanercept trials including 1,323 subjects (>1,500 subject years of treatment) (117) reported a serious infections rate of 2.19 (95% CI = 0.22–107.79) for the TNFi exposed compared with sulfasalazine exposed or placebo.

Based on this increased risk, specific recommendations for vaccination have been published for patients exposed to biologics and regardless of age, seasonal flu and pneumococcal vaccination are strongly recommended in these patients (123). Despite this, vaccination rates are far from optimal in this population: a cross-sectional study of 1,911 patients at risk among the 3,989 SpA included patients, only 332 (17.3%) had received a pneumococcal vaccination within the past 5 years, and 726 (38.0%) had received an influenza vaccination within the past 12 months (5). These results suggest that there remains an unmet need for improving infection prevention in SpA, particularly in high-risk cases such as patients exposed to biologics.

In summary, regarding infection risk, data are sparse compared with RA, but there appears to be no risk in the absence of TNFi exposure.

## Conclusion

Osteoporosis is a highly prevalent comorbidity in SpA, higher than the general population; however, risk of VF requires further evaluation, as current prevalence and incidence reported in the literature might have been over-estimated by the automate and semiautomate methods of evaluation of such VFs. Nevertheless, these findings support that measurement of BMD should be performed at least once during the disease course. Cardiovascular risk is also increased in this population, both due to an increase of the classic CVRF in these patients, but also due to the presence of inflammation. The role of NSAIDs in this increased risk needs further elucidation, but there is consensus for the need to encourage smoking cessation and to perform periodic evaluation of CV risk in these patients, particularly in case of treatment change. In the case of cancer, no increased risk inherent to SpA seems to exist. However, an increased neoplastic risk can occur due to SpA treatments, e.g., P-UVA; data on TNFi are controversial but no increased risk appears to exist in SpA, in contrast to RA. Furthermore, regarding infection risk, data are sparse compared with RA, but there appears to be no risk in the absence of TNFi exposure.

Regardless of the comorbidity, what is apparent is that a gap exists between recommendations for their management and implementation in clinical practice, suggesting that there is still room for improvement in this area. Systematic screening for these comorbidities should improve short and long-term outcomes in comorbid SpA patients, but to date the confirmatory data are lacking. Currently, ongoing studies should confirm this hypothesis.

## Author Contributions

All authors listed have made a substantial, direct, and intellectual contribution to the work and approved it for publication.

## Conflict of Interest Statement

The authors declare that the research was conducted in the absence of any commercial or financial relationships that could be construed as a potential conflict of interest.
